# Effects of *Baccharis dracunculifolia* DC on an Innovative Animal Model of Cardiometabolic Syndrome

**DOI:** 10.3390/pharmaceutics16111446

**Published:** 2024-11-12

**Authors:** Gustavo Ratti da Silva, Arianne Jung Kluck, Edilson Rodrigues Albuquerque, Lucas Pires Guarnier, Fernanda de Abreu Braga, Ester Pelegrini Silva, Karina Sposito Negrini, Juliana Aparecida Mendonça, Zilda Cristiani Gazim, Arquimedes Gasparotto Junior, João Tadeu Ribeiro-Paes, Francislaine Aparecida dos Reis Lívero

**Affiliations:** 1 Laboratory of Preclinical Research of Natural Products, Post-Graduate Program in Animal Science with Emphasis on Bioactive Products, Paranaense University, Umuarama 81531-980, Brazil; gustavoratti@gmail.com (G.R.d.S.); albuquerque.ed@gmail.com (E.R.A.); 2 Laboratory of Cardiometabolic Pharmacology, Federal University of Paraná (UFPR), Curitiba 81531-990, Brazil; ariannejung1@gmail.com; 3 Department of Genetic, Ribeirão Preto Medical School, University of São Paulo, Ribeirão Preto 14049-900, Brazil; lucasguarnier@usp.br; 4 Laboratory of Preclinical Research of Natural Products, Post-Graduate Program in Medicinal Plants and Phytotherapeutics in Basic Attention, Paranaense University, Umuarama 81531-980, Brazil; f.braga@edu.unipar.br (F.d.A.B.); ester.s@edu.unipar.br (E.P.S.); karina.negrini@edu.unipar.br (K.S.N.); 5 Chemistry Laboratory of Natural Products, Post-Graduate Program in Biotechnology Applied to Agriculture, Paranaense University, Umuarama 81531-980, Brazil; juliana.mendonca@edu.unipar.br; 6 Chemistry Laboratory of Natural Products, Post-Graduate Programs in Animal Science and Biotechnology Applied to Agriculture, Paranaense University, Umuarama 81531-980, Brazil; cristianigazim@prof.unipar.br; 7 Laboratory of Cardiovascular Pharmacology, Faculty of Health Sciences, Federal University of Grande Dourados, Dourados 79804-970, Brazil; arquimedesgasparotto@gmail.com; 8 Laboratory of Genetics and Cell Therapy (GenTe Cel), Department of Biotechnology, São Paulo State University, Assis 19806-900, Brazil; ribeiro.paes@unesp.br

**Keywords:** alecrim-do-campo, diabetes, dyslipidemia, metabolic syndrome, preclinical study, smoking, traditional medicine

## Abstract

**Background/Objective:** Cardiometabolic syndrome (CMS) is a complex clinical condition that encompasses metabolic dysregulation, cardiovascular disease, and diabetes risk factors. Worldwide, CMS is underdiagnosed, and its occurrence significantly increases cardiovascular morbimortality. Despite available pharmacological treatments, the approach is fragmented, and the associated clinical conditions are treated independently. This approach may be partially due to limited preclinical models to mimic the clinical conditions of CMS. Therefore, our study aims to present an innovative animal model of cardiometabolic syndrome and evaluate the effects of *Baccharis dracunculifolia* on the set of clinical alterations associated with the condition. **Methods:** Female Wistar rats were induced to develop diabetes, fed a cholesterol-enriched diet, and exposed to the smoke of 9 cigarettes/day for 6 weeks. During the last 2 weeks, the rats were treated with vehicle, *B. dracunculifolia* (30, 100, and 300 mg/kg), or a combination of simvastatin and insulin. At the end of the treatment, plasma lipid levels were measured, and the liver was analyzed histologically for hepatic lipid quantification and oxidative stress assessment. **Results:** Phytochemical analysis revealed seven phenolic acids and six flavonoids in the extract. *B. dracunculifolia* showed significant hepatoprotective effects, reducing AST and ALT levels and lowering both plasma and hepatic lipid levels. The extract also reversed hepatic steatosis and demonstrated antioxidant properties. **Conclusions:** These findings suggest that *B. dracunculifolia* may be a therapeutic option for the metabolic dysregulation present in CMS.

## 1. Introduction

Cardiometabolic syndrome has become a significant public health that encompasses a range of interrelated risk factors, including obesity, hypertension, type 2 diabetes, dyslipidemia, and insulin resistance, which together significantly increase the risk of cardiovascular diseases and metabolic disorders [1,2,3]. This syndrome presents a growing challenge to global public health, with its complexity lying in the synergistic interactions among these risk factors, leading to a cascade of detrimental effects on multiple organs, particularly the heart and blood vessels [4].

The pathogenesis of cardiometabolic syndrome involves intricate mechanisms, including chronic inflammation, oxidative stress, endothelial dysfunction, and alterations in lipid and glucose metabolism. Among the key drivers of these processes are diabetes, smoking, and dyslipidemia, which not only exacerbate cardiovascular risks but also contribute to the systemic metabolic imbalance that characterizes the syndrome. Understanding the interconnection between these risk factors is crucial for developing comprehensive therapeutic approaches [1,5].

Cardiometabolic syndrome affects more than one-quarter of the global adult population and up to 48.6% of individuals aged 60 years and over, contributing significantly to cardiovascular morbidity and mortality [6,7,8]. This alarming trend underscores the urgent need for comprehensive and effective preclinical models that replicate the complex, multifactorial nature of cardiometabolic syndrome, thus facilitating the development of targeted therapeutic interventions. Traditional animal models often fail to capture the full spectrum of cardiometabolic disturbances, as they tend to isolate individual risk factors rather than simulating the multifactorial nature of the disease. The need for innovative experimental models that mirror the complexity of human cardiometabolic syndrome is essential for advancing therapeutic strategies [5,9]. Such models allow for the assessment of interventions that target multiple metabolic pathways, reflecting the real-world conditions of the syndrome [9,10].

In recent years, there has been growing interest in exploring natural products with potential therapeutic benefits in the treatment of cardiometabolic conditions. Consequently, there is increasing interest in exploring alternative and complementary therapies, particularly those derived from natural products known for their safety profiles and containing bioactive compounds with potential therapeutic effects [11,12,13].

A species with potential popular use but few ethnopharmacological studies is *Baccharis dracunculifolia*, a shrub belonging to the Asteraceae family, commonly known in Brazil as ‘alecrim-do-campo’ or ‘vassourinha’ [14]. This plant is commonly used in the form of tea to treat gastric diseases, inflammation, and liver disorders [3]. Preclinical studies conducted with crude extracts of *B. dracunculifolia* have demonstrated various biological effects, such as anti-inflammatory [15], antiulcerogenic [16], antibacterial [17], antioxidant [18], anticancer [19], immunomodulatory [20], antigenotoxic, and antimutagenic properties [21].

The present study aims to investigate the therapeutic effects of *B. dracunculifolia* extract in an innovative animal model that simulates the combined conditions of diabetes, dyslipidemia, and smoking-induced metabolic dysfunctions. By addressing the multifactorial aspects of the syndrome, this study seeks to evaluate the plant’s potential in modulating key metabolic parameters, thereby contributing to the development of novel strategies for managing cardiometabolic diseases.

## 2. Materials and Methods

### 2.1. Preparation of Baccharis dracunculifolia Extract and Phytochemical Composition

The aerial parts (leaves and flowers) of *Baccharis dracunculifolia* were collected in a rural area of the municipality of Maria Helena, Paraná, Brazil (23°39′49.7″ S and 53°18′18.0″ W). The plant material was dried at room temperature (32 °C). Then, it was ground to a particle size of 850 µm. The resulting powder was subjected to a dynamic maceration process with solvent renewal using 96% ethanol (*v*/*v*) until the plant material was exhausted. The filtrate was then concentrated under reduced pressure in a rotary evaporator (Tecnal^®^, model TE-211, Piracicaba, Brazil) at 40 °C, until obtaining the crude ethanolic extract of *B. dracunculifolia*, which was subsequently stored at −20 °C. The analysis of the extracts was carried out using ultra-high performance liquid chromatography coupled with high resolution mass spectrometry (UHPLC-ESI-QTOF-MS/MS) following the methodology described by Silva et al. [22]. The chemical analysis was biodirected toward the investigation of phenolic compounds, and identification was performed as proposed in review studies on the genus *Baccharis* [23,24,25] by calculating the mass error and comparing the results with data from MassBank (http://www.massbank.jp/, accessed on 25 May 2021) and the Human Metabolome Database (http://www.hmdb.ca/, accessed on 25 May 2021).

### 2.2. Pharmacological Studies

#### 2.2.1. Animals

The experimental model was developed using female Wistar rats, weighing between 200 and 250 g, obtained from the Central Animal Facility of the Federal University of Grande Dourados. The choice of female rats was based on evidence suggesting a greater susceptibility to dyslipidemia under dietary modification, ensuring consistent model induction [26]. The animals were housed in the Animal Facility of the Neuroscience Laboratory at Paranaense University (UNIPAR), provided free access to liquid and solid diet, and maintained under controlled environmental conditions (temperature: 20 ± 2 °C; relative humidity: 50 ± 10%; light/dark cycle: 12 h; and environmental enrichment). After randomization, the rats were divided into 6 experimental groups (*n* = 6–8). They were weighed weekly on an analytical balance. The entire experimental protocol was approved by the Ethics Committee on Animal Experimentation of UNIPAR under protocol number 1003/2022. All regulations and recommendations of the National Council for the Control of Animal Experimentation (CONCEA, Brazil) were followed in accordance with the guidelines of the Animal Research Reporting of In Vivo Experiments [27], aiming at animal welfare and a reduction in the number of animals used for experimentation.

#### 2.2.2. Experimental Design and Treatments

The model was established based on the associations among diabetes, dyslipidemia, and smoking. For the induction of diabetes, the animals received streptozotocin (30 mg/kg, i.p.) diluted in citrate buffer (10 mM, pH 4.5) as described by Souza et al. [28] and Vit et al. [29]. The rats were considered diabetic when their blood glucose levels were equal to or greater than 250 mg/dL.

Dyslipidemia was induced in the animals by providing a diet enriched with 0.5% cholesterol. This diet was prepared by mixing 150 g of standard rodent chow with one egg yolk and 13.5 mL of corn oil. The blend was combined with water, baked at 50 °C for 36 h, and stored in vacuum-sealed bags. Each 150 g portion of this diet contained 225 mg of cholesterol, 1.8 g of saturated fat, 2.16 g of monounsaturated fats, and 0.72 g of polyunsaturated fats [30].

The animals were exposed to 9 cigarettes (containing 0.8 mg nicotine, 10 mg tar, and 10 mg carbon monoxide) for 1 h per day, 5 days a week. Basal animals underwent the same conditions without smoke exposure. The setup included a smoke capture and distribution system adapted for smoking studies, with five cubic metal cages (24 cm × 17 cm × 15 cm) that each held three animals. These cages were spaced equally within a sealed acrylic box (70 cm × 70 cm × 20 cm). Cigarette smoke was drawn through an external holder using an electric pump with an automatic timer, allowing intermittent air intake to prevent asphyxiation. Inside the box, the tubing branched out to direct smoke into each cage, dispersing it evenly across the chamber [30].

During the last 2 weeks of the study, the rats received oral treatments via gavage with either a vehicle (filtered water, 1 mL/kg, serving as the negative control [C−]) or varying doses of *B. dracunculifolia* extract (30, 100, and 300 mg/kg). The positive control group (SIM + INSU) was treated with simvastatin (2.5 mg/kg, orally) and insulin (6 IU, subcutaneously). The doses of both the drugs and the extract used in this study were determined according to the guidelines proposed by Barbosa et al. [31], Zago et al. [32], and Auth et al. [33].

**Figure 1:**
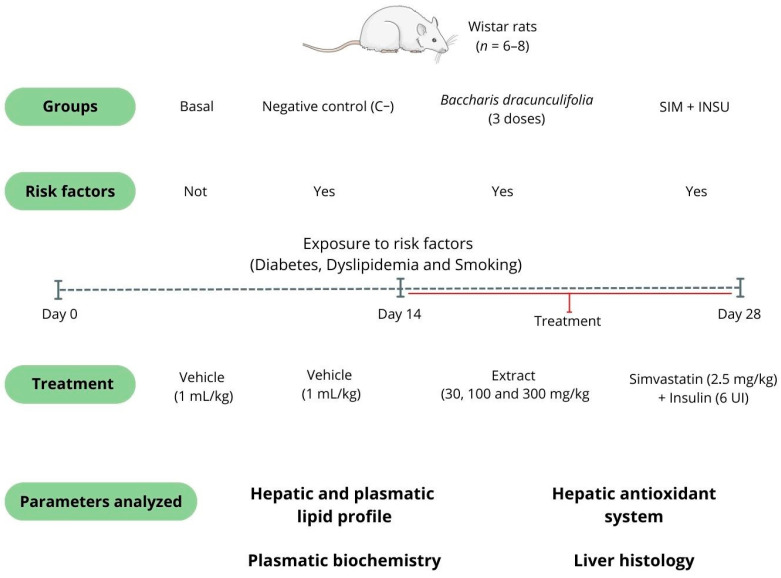
The model of fatty liver disease associated with metabolism was generated by inducing diabetes, dyslipidemia, and smoking conditions in Wistar rats. The study lasted for 4 weeks and consisted of a baseline group [normoglycemic rats not exposed to risk factors and treated with vehicle (filtered water, 1 mL/kg, *n* = 6)] and five other groups exposed to risk factors and treated orally (gavage) in the last two experimental weeks with vehicle (filtered water, 1 mL/kg, negative control [C−], *n* = 8), *Baccharis dracunculifolia* extract (30, 100, and 300 mg/kg, *n* = 8), and simvastatin (2.5 mg/kg) + insulin (6 IU, s.c., positive control group [SIM + INSU], *n* = 8). At the end of the treatment, the lipid-lowering and hepatoprotective effects of *B. dracunculifolia* were analyzed.

The selection of *B. dracunculifolia* dosages aimed to investigate potential dose–response relationships and to identify the optimal therapeutic window for this phytotherapeutic agent. Previous studies involving *Baccharis* spp. [28,31,34,35] have shown that low-to-moderate doses often yield maximal efficacy due to the biphasic effects commonly associated with natural products. By including this range of dosages, we were able to assess both subtherapeutic and potentially saturating doses, clarifying the most effective dose in terms of metabolic and hepatic effects. Additionally, a baseline group was included, consisting of rats that were not exposed to the risk factors and were treated solely with the vehicle (filtered water, 1 mL/kg; see Figure 1).

#### 2.2.3. Euthanasia, Sample Collection, and Biochemical Analysis

At the end of the four-week period, following a 12 h fast, the animals were euthanized by deepening anesthesia in an isoflurane (1–3%) saturation chamber. Blood was then collected via decapitation, with glucose levels immediately measured using a blood glucose meter (Roche^®^, model Accu-Chek Active, Mannheim, Germany). The remaining blood was centrifuged at 1500× *g* for 10 min, and the plasma was stored at −80 °C for subsequent analysis. Plasma levels of cholesterol, triglycerides, glucose, aspartate aminotransferase (AST), and alanine aminotransferase (ALT) were measured using colorimetric methods on a semi-automatic analyzer (Global Analyzer^®^, model GTA-300, Calgary, AB, Canada). After blood collection, abdominal laparotomy was performed to collect the liver, which was washed with saline and weighed on an analytical balance. A sample of liver tissue was quickly collected and frozen in liquid nitrogen for evaluation of the tissue redox state. Another sample of the organ was stored for histological analyses.

#### 2.2.4. Measurement of Hepatic Cholesterol and Triglycerides

Hepatic cholesterol and triglycerides determination was performed following the methodology described by Lívero et al. [34]. For this purpose, liver samples were lyophilized, subjected to extraction with hexane (solvent, 1:10 [tissue:solvent]), and heated in an oven (80 °C, 12 h). After this period, the supernatant was transferred to a new container and allowed to evaporate naturally, with this extraction step being repeated three times. The percentage of hepatic lipids was calculated using the formula [lipids (%) = 100 × (final weight of the flask/initial weight of the flask)/0.3 g]. Additionally, the resulting material from the extractions was resuspended in chloroform (1 mL) and isopropanol (2 mL) to determine the levels of cholesterol and triglycerides by colorimetric enzymatic technique on a semi-automatic analyzer (Global Analyzer^®^, model GTA-300).

#### 2.2.5. Investigation of the Hepatic Antioxidant System

To evaluate the hepatic antioxidant potential of *B. dracunculifolia*, liver samples were initially homogenized in a potassium phosphate buffer solution (0.1 M, pH 6.5) at a 1:10 dilution. Next, 100 μL of the homogenate was separated and combined with 80 μL of 12.5% trichloroacetic acid. The solution was then vortexed and centrifuged at 6000 rpm for 15 min at 4 °C to measure reduced glutathione (GSH) levels, following the method outlined by Sedlak and Lindsay [36]. The remaining homogenate was further centrifuged at 9700 rpm for 20 min at 4 °C to assess superoxide dismutase (SOD) activity and lipid peroxidation (LPO) levels, according to protocols by Gao et al. [37] and Jiang et al. [38], respectively. Each assay was performed in triplicate, and results were normalized per gram of tissue.

#### 2.2.6. Histopathological Analysis

The hepatic tissue was fixed in 10% buffered formalin (1800 mL distilled water, 200 mL formaldehyde, 8 g monobasic sodium phosphate, and 13 g dibasic sodium phosphate). After fixation, the sample was dehydrated with alcohol and xylene, and subsequently embedded in paraffin. Sections were cut at a thickness of 6 μm and stained with hematoxylin and eosin for assessment of general morphology and Sudan Black for lipid visualization. For triglyceride-specific staining, an additional liver sample underwent a distinct preparation. The sample was gradually saturated in sucrose solutions of increasing concentrations (10%, 20%, and 30%), with each concentration maintained for 24 h. Following saturation, the sample was embedded in Tissue Tek (O.C.T. Sakura, Torrance, CA, USA) and rapidly frozen using liquid nitrogen. Frozen sections, cut at a thickness of 6 μm, were then stained with Sudan Black, as adapted from the methodology of Lívero et al. [34]. The slides were examined using an optical microscope (Leica^®^, model DM 2500, Wetzlar, Germany) to assess structures and cellular abnormalities. Hepatic lesions were classified on a scale from 0 to 3 according to the percentage of tissue affected by multiple risk factors (grade 0: less than 5% occurrence; grade 1: 6–33% occurrence; grade 2: 34–66% occurrence; and grade 3: 67–100% occurrence), following the classification by Lívero et al. [34] and Mendes et al. [39]. To enhance the robustness of our histological analysis, the number of fields assessed for each animal was standardized. Specifically, we analyzed five fields per animal to provide a comprehensive evaluation of the histological findings.

### 2.3. Blinding Clarification and Statistical Analysis

To address potential bias, the study was conducted using a blind approach. Investigators responsible for data collection and analysis were unaware of the group assignments throughout the experimental process and data analysis. This blinding was strictly maintained until the conclusion of data analysis to minimize any potential bias. The data were first analyzed for homogeneity of variance and normality of distribution. One-way ANOVA, followed by Bonferroni’s post-test, was used. The level of significance adopted was 95% (*p* < 0.05). GraphPad Prism^®^ version 9.0 was used for statistical analysis and graph preparation.

## 3. Results

### 3.1. Phytochemical Analysis

The phytochemical investigation indicated the presence of seven phenolic acids (quinic acid, chlorogenic acid, 4-hydroxybenzoic acid, caffeic acid, *p*-coumaric acid, ferulic acid, and protocatechuic acid) and six flavonoids (isoquercetin, quercetin, isokaempferide, kaempferol, 3-methoxy-quercetin, and apigenin). The compounds, theoretical mass *m*/*z* [M-H], experimental mass m/z [M-H], and retention time (min) are described in Table 1.

### 3.2. Baccharis dracunculifolia Did Not Alter the Body Weight and Chow Consumption of Rats

The average body weight of the basal group did not show a significant difference between the initial weight (274.30 ± 1.87 g) and the final weight after four weeks (287.90 ± 3.58 g). Also, no difference was observed in the average feed consumption between the experimental weeks. No statistical differences were found when analyzing the body weight gain or feed consumption of the groups exposed to risk factors and treated with different doses of *B. dracunculifolia* or SIM + INSU throughout the four weeks of the experiment (Table 2).

### 3.3. Baccharis dracunculifolia Exhibit a Median Hypoglycemic Effect and Demonstrated Significant Hepatoprotective Properties

**Figure 2:**
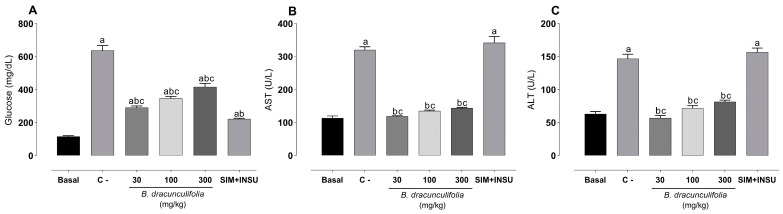
Plasma levels of (**A**) glucose, (**B**) aspartate aminotransferase, and (**C**) alanine aminotransferase in normoglycemic, non-dyslipidemic, and non-smoker Wistar rats (basal group, *n* = 6) and diabetic, dyslipidemic, and smoker Wistar rats that were treated with vehicle (negative control [C−], *n* = 8), *Baccharis dracunculifolia* (30, 100, and 300 mg/kg, *n* = 8), and simvastatin (2.5 mg/kg) + insulin (6IU; SIM + INSU, *n* = 8). The data are expressed as mean ± SEM. ^a^ *p* < 0.05, vs. basal; ^b^ *p* < 0.05, vs. C−; ^c^ *p* < 0.05, vs. SIM + INSU (one-way ANOVA followed by Bonferroni post hoc test).

After diabetes induction, a significant increase in blood glucose levels was noted in the C− group (636.20 ± 32.38 mg/dL) compared to the basal group (115.20 ± 5.07 mg/dL). The administration of SIM + INSU resulted in a decrease in blood glucose levels (222.00 ± 4.09 mg/dL), while treatment with *B. dracunculifolia* partially reversed these alterations (Figure 2A). Diabetes, dyslipidemia, and smoking led to a significant rise of approximately 132.53% in ALT and 182.92% in AST levels compared to the basal group (63.00 ± 3.73 U/L and 113.00 ± 6.71 U/L, respectively). However, the administration of 30 mg/kg *B. dracunculifolia* extract completely reversed the elevation in AST and ALT levels. Treatment with 100 and 300 mg/kg *B. dracunculifolia* extract partially reversed these alterations, and the combination of SIM+INS was ineffective (Figure 2B,C).

### 3.4. Baccharis dracunculifolia Extract Exerted Lipid-Lowering Effects

**Figure 3:**
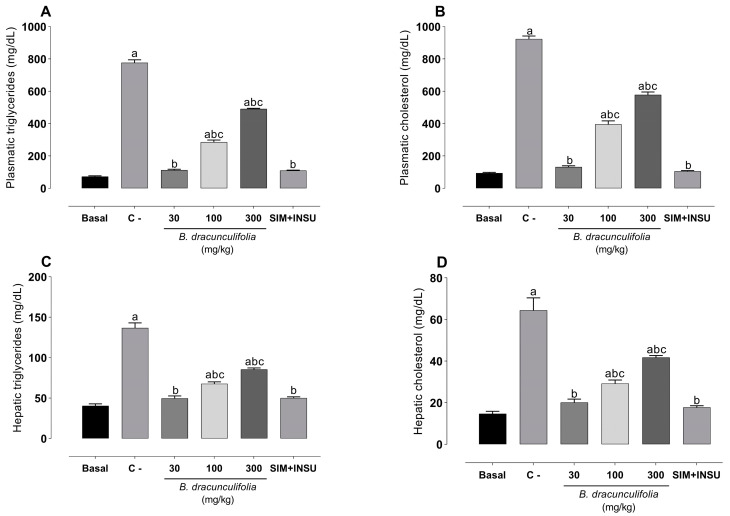
Plasma levels of (**A**) triglycerides and (**B**) cholesterol as well as hepatic levels of (**C**) triglycerides and (**D**) cholesterol in normoglycemic, non-dyslipidemic, and non-smoker Wistar rats (basal group, *n* = 6) and diabetic, dyslipidemic, and smoker Wistar rats that were treated with vehicle (negative control [C−], *n* = 8), *Baccharis dracunculifolia* (30, 100, and 300 mg/kg, *n* = 8), and simvastatin (2.5 mg/kg) + insulin (6IU; SIM + INSU, *n* = 8). n = 8/group. The data are expressed as mean ± SEM. ^a^ *p* < 0.05, vs. basal; ^b^ *p* < 0.05, vs. C−; ^c^ *p* < 0.05, vs. SIM + INSU (one-way ANOVA followed by Bonferroni post hoc test).

The presence of diabetes, dyslipidemia, and smoking significantly elevated plasma levels of triglycerides (776.80 ± 18.14 mg/dL, Figure 3A) and cholesterol (924.00 ± 18.11 mg/dL, Figure 3B) compared to the basal group (72.50 ± 4.58 mg/dL and 94.17 ± 4.42 mg/dL, respectively). These risk factors also induced a substantial increase in hepatic triglyceride (237.36%, Figure 3C) and cholesterol (339.19%, Figure 3D) levels compared to the basal group (40.52 ± 2.32 mg/dL and 14.67 ± 1.25 mg/dL, respectively). However, treatment with 30 mg/kg *B. dracunculifolia* extract and SIM + INSU completely reversed these alterations in both plasma and liver. Treatment with 100 and 300 mg/kg *B. dracunculifolia* extract partially restored triglyceride and cholesterol levels in plasma and liver (Figure 3).

### 3.5. Baccharis dracunculifolia Reversed Hepatocellular Alterations

**Figure 4:**
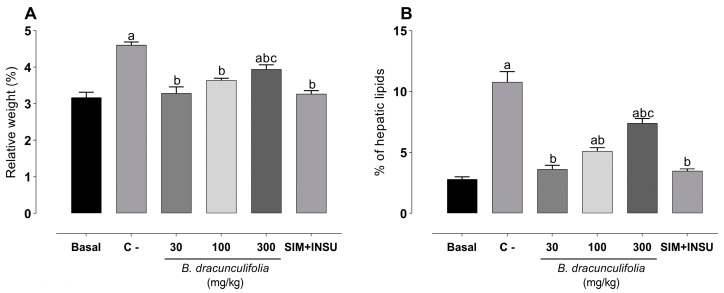
Relative (%) hepatic weight (**A**) and % of hepatic lipids (**B**). The study involved normoglycemic, non-dyslipidemic, and non-smoker Wistar rats (basal group, *n* = 6) as well as diabetic, dyslipidemic, and smoker Wistar rats treated with vehicle (negative control [C−], *n* = 8), *Baccharis dracunculifolia* (30, 100, and 300 mg/kg, *n* = 8), and simvastatin + insulin (SIM + INSU, *n* = 8). The data are shown as mean ± SEM. ^a^ *p* < 0.05, vs. basal; ^b^ *p* < 0.05, vs. C−; ^c^ *p* < 0.05, vs. SIM + INSU (one-way ANOVA followed by Bonferroni post hoc test).

The results shown in Figure 4 reveal significant findings regarding liver weight, lipid percentage, and histopathological analyses. First, the relative liver weight in the C− group exhibited a significative increase (4.60 ± 0.08%) compared to the basal group (3.16 ± 0.14%). However, treatment with *B. dracunculifolia* (30 and 100 mg/kg) effectively normalized this parameter (Figure 4A), while a dose of 300 mg/kg showed intermediate results. Additionally, risk factors significantly elevated the hepatic lipid percentage by 283.62% in the C− group compared to the basal group (2.81 ± 0.19%). Conversely, treatment with 30 mg/kg of *B. dracunculifolia* extract successfully reversed these changes (Figure 4B), with doses of 100 mg/kg and 300 mg/kg showing intermediate improvement.

**Figure 5:**
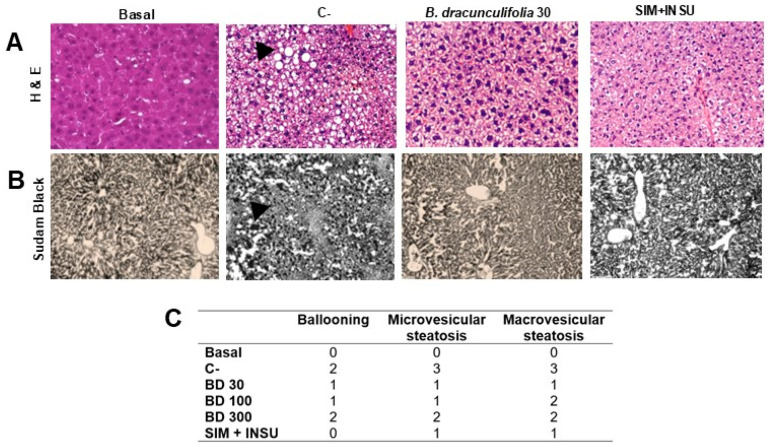
Hepatic histopathological analysis with hematoxylin/eosin (**A**), Sudan Black (**B**), and grades of liver injury (**C**). The study involved normoglycemic, non-dyslipidemic, and non-smoker Wistar rats (basal group, *n* = 6) as well as diabetic, dyslipidemic, and smoker Wistar rats treated with vehicle (negative control [C−], *n* = 8), *Baccharis dracunculifolia* (30, 100, and 300 mg/kg, *n* = 8), and simvastatin + insulin (SIM + INSU, *n* = 8). In the histopathological analysis, black arrows indicate hepatic fatty degeneration (steatosis). The images were taken at 40× magnification.

To confirm the presence of steatosis and cellular alterations, liver samples were stained with hematoxylin and eosin and Sudan Black, revealing macroscopic liver alterations that indicate the successful induction of hepatic injury (Figure 5A,B). In the basal group, the absence of ballooning and microvesicular and macrovesicular steatosis was observed. In contrast, group C− exhibited the highest grades across all three evaluated categories, with grade 2 ballooning, grade 3 microvesicular steatosis, and grade 3 macrovesicular steatosis. Groups treated with varying doses of *B. dracunculifolia* showed variations in the degrees of hepatic injury: grade 1 ballooning and steatosis at 30 mg/kg; grade 1 ballooning, grade 1 microvesicular steatosis, and grade 2 macrovesicular steatosis at 100 mg/kg; and grade 2 ballooning and grade 2 steatosis at 300 mg/kg. Treatment with SIM + INSU resulted in grade 1 ballooning and steatosis (Figure 5C).

### 3.6. Effects of Baccharis dracunculifolia on Hepatic Redox State

**Figure 6:**
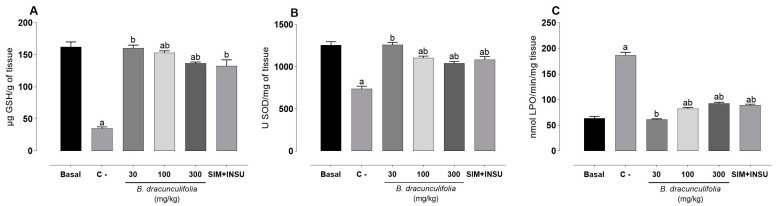
Reduced glutathione (**A**), superoxide dismutase (**B**), and lipoperoxidation (**C**). The study involved normoglycemic, non-dyslipidemic, and non-smoker Wistar rats (basal group, *n* = 6) as well as diabetic, dyslipidemic, and smoker Wistar rats treated with vehicle (negative control [C−], *n* = 8), *Baccharis dracunculifolia* (30, 100, and 300 mg/kg, *n* = 8), and simvastatin + insulin (SIM + INSU, *n* = 8). The data are shown as mean ± SEM. ^a^ *p* < 0.05, vs. basal; ^b^ *p* < 0.05, vs. C− (one-way ANOVA followed by Bonferroni post hoc test).

The oxidative stress analysis revealed significant variations among the experimental groups for the three parameters: GSH, SOD, and LPO. The basal group exhibited the highest levels of GSH (162.40 ± 7.68 µg GSH/g of tissue) and SOD (1258 ± 39.80 U SOD/mg of tissue) and the lowest levels of LPO (63.70 ± 3.60 mmol LPO/min/g of tissue). The C− group showed a sharp reduction in GSH (35.16 ± 2.42 µg GSH/g of tissue) and SOD (739.40 ± 30.80 U SOD/mg of tissue) and a substantial increase in LPO (186.90 ± 5.58 mmol LPO/min/g of tissue). Among the treated groups, treatment with 30 mg/kg *B. dracunculifolia* extract maintained GSH (160.60 ± 4.40 µg GSH/g of tissue) and SOD (1260 ± 29.45 U SOD/mg of tissue) levels close to basal levels, with low LPO levels (61.25 ± 1.34 mmol LPO/min/g of tissue). Higher doses of *B. dracunculifolia* extract (100 and 300 mg/kg) and the combination of SIM + INSU resulted in partially reduced GSH and SOD levels and increased LPO levels (Figure 6).

## 4. Discussion

Cardiometabolic syndrome is a multifactorial condition that requires an integrative approach to management [40,41]. Developing innovative and comprehensive models is essential to advancing our understanding and treatment of this complex syndrome. Our study contributes to this goal, showing that *B. dracunculifolia* effectively targets key metabolic disturbances associated with cardiometabolic syndrome, including hyperglycemia, dyslipidemia, and hepatic and cardiovascular dysfunction. Our findings reveal the multifaceted therapeutic potential of *B. dracunculifolia*, which stands out for its ability to modulate glycemic control, lipid profiles, hepatic markers, and oxidative stress—addressing the interconnected pathways that drive disease progression. *B. dracunculifolia* not only impacts isolated components of the syndrome but also appears to offer an integrative therapeutic approach by simultaneously influencing multiple risk factors. This holistic effect is especially valuable given the limitations of conventional treatments, which often target individual aspects of the disease without addressing the full range of metabolic dysfunctions. These promising findings underscore the need for further investigation into the underlying mechanisms and potential clinical applications of *B. dracunculifolia* as a complementary therapy for managing cardiometabolic syndrome.

Our study indicates that the therapeutic potential of *B. dracunculifolia* in managing cardiometabolic syndrome may be largely attributed to its complex array of bioactive compounds, including flavonoids and phenolic acids. These compounds possess a range of biological activities that may act synergistically, enhancing the plant’s overall therapeutic effect. Flavonoids such as quercetin and rutin are well-documented for their antioxidant, anti-inflammatory [42,43,44], and lipid-lowering properties [45,46], which likely contribute significantly to reducing oxidative stress [47,48,49] and improving lipid profiles in the context of cardiometabolic syndrome. The reductions in lipid peroxidation and improvements in antioxidant markers, such as GSH and SOD observed in our study, support this mechanism. Additionally, phenolic acids like caffeic and p-coumaric acids enhance *B. dracunculifolia*’s bioactivity, promoting cardiovascular and metabolic health through their vasodilatory and lipid-regulating effects [50,51]. These compounds may interact with flavonoids, potentially amplifying the extract’s therapeutic effects through synergistic actions that target multiple cardiometabolic risk factors simultaneously [52].

The dose-dependent response observed with *B. dracunculifolia* extract further underscores the complexity of its therapeutic action. While lower doses completely reversed hyperlipidemia and liver enzyme elevations, higher doses showed only partial effects. This phenomenon could be attributed to saturation, synergistic or antagonistic effects, or the presence of different bioactive compounds at varying concentrations, each exerting distinct biological activities [53,54]. This warrants further investigation into the pharmacokinetics and pharmacodynamics of *B. dracunculifolia* to better understand its therapeutic window and optimize its dosing regimen.

The lack of results with the highest dose of *B. dracunculifolia* can be explained by the hormetic effect. This phenomenon is commonly observed when working with crude plant extracts, which contain various secondary metabolites. These compounds may act independently, synergistically, or even antagonistically on different biological targets. Typically, a substance—or a combination of substances—exhibits a dose at which its maximal effect is achieved. Beyond this dose, increasing the amount yields no further therapeutic benefit, a point referred to as the plateau. This type of dose–response curve is often observed because the biological tissue or function in question has already reached its maximum response.

The partial reversal of hyperglycemia by *B. dracunculifolia* suggests that this extract may contain bioactive compounds capable of modulating glucose metabolism. The hypoglycemic effects of *B. dracunculifolia* are corroborated by studies on other medicinal plants with similar properties. Several preclinical studies have demonstrated the hypoglycemic effects of plant flavonoids [55,56,57]. These results emphasize the role of natural products in managing diabetes and its complications. This is particularly significant in the context of cardiometabolic syndrome, where insulin resistance and hyperglycemia are pivotal contributors to disease progression.

The protective effects of *B. dracunculifolia* on cardiovascular and metabolic health, evidenced by the improvement in liver enzymes and metabolic markers, suggest its role in modulating oxidative stress, which is central to the pathophysiology of cardiometabolic syndrome. The normalization of markers such as GSH, SOD, and LPO levels following treatment with *B. dracunculifolia* indicates its capacity to mitigate oxidative stress, further supporting its use in addressing the oxidative burden linked to this syndrome [58]. The analysis of redox status revealed significant variations among the experimental groups for the GSH, SOD, and LPO parameters. The C− group showed substantial reductions in GSH and SOD levels, accompanied by a marked increase in LPO, indicative of pronounced oxidative stress associated with the studied live condition. Treatment with *B. dracunculifolia* extract demonstrated protective effects against hepatic oxidative stress. This treatment maintained GSH and SOD levels close to those of the basal group and reduced LPO levels, indicating the extract’s ability to preserve liver antioxidant defenses and mitigate lipid peroxidation associated with the model studied. These findings are consistent with existing literature on the variable effects of botanical extracts of *Baccharis* genus on oxidative stress, depending on dosage and experimental conditions [28,31,34,35,39,59,60,61].

Elevated levels of ALT and AST enzymes are markers of hepatic injury, and their reduction indicates a protective effect on liver tissue. In the context of the *Baccharis* genus, Barbosa et al. [31] demonstrated that *B. trimera* extract reduced ALT and AST levels in rats exposed to smoking, dyslipidemia, and diabetes. Additionally, *B. trimera* exhibited a hepatoprotective effect in a mouse model of alcoholic liver disease. Lívero et al. [34] further reported that treatment with *B. trimera* reversed the increase in ALT and AST levels in mice exposed to ethanol and a low-protein diet, compared to those treated with a vehicle. In this study, the hepatoprotective effects of *B. dracunculifolia*, evidenced by the normalization of ALT and AST levels, are noteworthy. The extract’s ability to mitigate liver damage induced by combined metabolic risk factors suggests its potential role in modulating inflammation and oxidative stress, which are critical pathways in the progression of metabolic associated fatty liver disease (MAFLD), a condition that has been considered the hepatic manifestation of metabolic syndrome [62].

The ineffectiveness of the combination of SIM + INS in normalizing liver enzymes suggests that standard pharmacological treatments may not address all the pathological mechanisms involved in MAFLD. This finding is supported by Nassir [63], who emphasized the limitations of conventional treatments in addressing the multifactorial nature of MAFLD and the need for alternative therapeutic approaches. This highlights the need for alternative or complementary therapies that can target the disease’s multifactorial nature. *B. dracunculifolia*’s broad spectrum of biological activities, including anti-inflammatory, antioxidant, and immunomodulatory effects, positions it as a promising candidate for such therapies.

Simvastatin is a widely used lipid-lowering agent effective in managing dyslipidemia associated with cardiometabolic conditions. It functions by inhibiting HMG-CoA reductase, the enzyme responsible for cholesterol synthesis in the liver, resulting in decreased levels of low-density lipoprotein cholesterol and triglycerides [64]. Simvastatin was selected as a control agent in our study due to its well-established hypolipemiant effects, providing a reliable comparative standard for evaluating the potential therapeutic effects of *B. dracunculifolia*. In addition to simvastatin, we also employed insulin as part of the positive control regimen. Insulin plays a critical role in glucose metabolism and is often used to manage hyperglycemia in patients with diabetes, which is a common comorbidity in cardiometabolic disorders [65]. The combination of simvastatin and insulin serves as a robust positive control, allowing us to assess the efficacy of *B. dracunculifolia* in comparison to established therapeutic approaches.

Srikanth and Deedwania [66] highlighted the importance of managing dyslipidemia in patients with diabetes and metabolic syndrome. The significant elevation in plasma and hepatic triglyceride and cholesterol levels in the C− group reflects the dyslipidemia commonly associated with metabolic syndrome. The normalization of these lipid levels with 30 mg/kg *B. dracunculifolia* extract and SIM+INS indicates the efficacy of these treatments in managing dyslipidemia. This effect is crucial as elevated triglycerides and cholesterol contribute to the progression of liver steatosis and subsequent liver injury. The partial restoration with higher doses of *B. dracunculifolia* suggests that while the extract is effective, its lipid-lowering capacity might be influenced by the dosage.

The increase in relative liver weight and hepatic lipid percentage in the C− group indicates the successful induction of liver steatosis, a hallmark of MAFLD. The normalization of liver weight and lipid content with 30 mg/kg *B. dracunculifolia* treatment further supports its hepatoprotective effects. Histopathological analysis revealed significant liver alterations in the C− group, including steatosis, which were reversed by *B. dracunculifolia* treatment. This finding is critical as it confirms the macroscopic and microscopic protective effects of the extract against hepatic injury.

In our study, the weight stability observed in the C− group despite significant hyperglycemia raises interesting considerations regarding metabolic adaptations and the temporal aspects of diabetes induction. Typically, sustained hyperglycemia in diabetes models leads to weight loss due to increased protein and fat catabolism associated with insulin deficiency and poor glycemic control [67]. However, in this study, the diabetes induction period was relatively short (four weeks), which may have been insufficient to trigger the marked weight loss often seen in chronic hyperglycemic states. Furthermore, the controlled dietary conditions in this model provided consistent calorie intake, which may have mitigated the typical weight fluctuations observed in prolonged diabetic states. Another plausible explanation for this stability could be attributed to adaptive metabolic compensations [68]. Acute or sub-chronic models of diabetes, particularly when induced in a well-nourished and monitored animal cohort, may prompt compensatory mechanisms in peripheral tissues to temporarily preserve body weight. This adaptation could involve an upregulation of alternative metabolic pathways, such as increased reliance on lipid oxidation and amino acid catabolism, which may contribute to maintaining baseline body weight in the short term [69,70]. Additionally, while diabetes and associated metabolic disturbances lead to significant weight changes over time, these effects may not manifest fully within the timeframe used here. Future studies could extend the induction period and explore additional metabolic markers, such as ketone body levels and proteolysis indicators, to assess whether weight stability persists under prolonged hyperglycemia and if other compensatory mechanisms are activated. These insights could provide a more comprehensive understanding of the complex metabolic adaptations in early diabetic states and enhance the translational relevance of this model for studying cardiometabolic syndrome.

The study underscores the potential of *B. dracunculifolia* as a natural therapeutic agent for cardiometabolic syndrome, capable of addressing multiple facets of the disease, including hyperglycemia, dyslipidemia, and hepatic injury. Its efficacy at a lower dose suggests a favorable safety profile and encourages further research to optimize dosing strategies. Furthermore, the traditional use of *B. dracunculifolia* in treating various ailments supports its safety and efficacy; however, rigorous clinical trials are necessary to confirm these benefits in patients. The ethnopharmacological background provides a rich context for understanding how traditional knowledge can inform modern therapeutic strategies.

## 5. Conclusions

This study highlights the significant therapeutic potential of *Baccharis dracunculifolia* extract in alleviating the effects of cardiometabolic syndrome induced by diabetes, dyslipidemia, and smoking. The extract exhibited hypoglycemic properties and robust hepatoprotective effects, normalizing liver enzyme levels and lipid profiles. *B. dracunculifolia* shows promise as a complementary or alternative natural therapy, warranting further investigation to elucidate its molecular mechanisms, optimize dosing, and validate its clinical efficacy and safety.

## Figures and Tables

**Table 1:** Chemical composition of crude extract of *Baccharis dracunculifolia* aerial parts using UHPLC-ESI-QTOF-MS/MS.

Compounds	Theoretical Mass *m*/*z* [M-H]	Experimental Mass *m*/*z* [M-H]	Retention Time (min)	Error
Phenolic acids				
Quinic acid	191.05	191.05	0.84	−3.66
Chlorogenic acid	353.08	353.08	4.25	0
4-Hydroxybenzoic acid	137.02	137.02	4.10	−5.11
Caffeic acid	179.03	179.03	4.15	4.47
*p*-Coumaric acid	163.03	163.03	4.60	−4.29
Ferulic acid	193.04	193.05	5.51	−3.62
Protocatechuic acid	153.01	153.01	1.28	−3.92
Flavonoids				
Isoquercetin	463.08	463.08	4.40	2.59
Quercetin	301.03	301.03	5.16	0.33
Isokaempferide	299.05	299.05	5.45	−2.01
Kaempferol	285.03	285.03	5.48	0
3-Methoxy-quercetin	315.05	315.05	5.51	0.32
Apigenin	269.04	269.04	5.43	−0.74

**Table 2:** Body weight and food consumption of rats exposed to diabetes, smoking, and dyslipidemia and treated with *Baccharis dracunculifolia*.

Groups	Initial	At 2 Weeks		At 4 Weeks	
	Body Weight (g)	Body Weight (g)	Chow Consumption (g)	Body Weight (g)	Chow Consumption (g)
Basal	274.30 ± 1.87	283.70 ± 3.55	108.90 ± 2.93	287.90 ± 3.58	101.60 ± 4.98
C−	244.30 ± 5.57	245.40 ± 7.94	135.90 ± 3.23	254.50 ± 6.53	126.4 ± 6.43
BD 30	240.30 ± 2.71	233.80 ± 5.83	142.80 ± 5.06	230.30 ± 10.53	136.4 ± 4.88
BD 100	227.30 ± 4.25	228.90 ± 2.77	128.30 ± 8.57	230.40 ± 6.51	132.9 ± 8.53
BD 300	205.90 ± 7.36	223.10 ± 7.35	112.20 ± 3.72	231.10 ± 8.77	103.7 ± 5.10
SIM + INSU	241.90 ± 5.13	246.50 ± 4.71	133.80 ± 7.16	287.00 ± 4.98	153.60 ± 1.17

Values are presented as mean ± SEM (*n* = 6–8). Feed consumption in grams (g) per week. Legend: Basal, normoglycemic rats; C−, negative control; BD30, BD 100, and BD 300, *B. dracunculifolia* at doses of 30, 100, and 300 mg/kg, respectively; SIM + INSU, simvastatin (2.5 mg/kg) plus insulin (6 IU).

## Data Availability

The original contributions presented in the study are included in the article, further inquiries can be directed to the corresponding author.
